# The Off-Label Use of Antineoplastics in Oncology Is Limited But Has Notable Scientific Support in a University Hospital Setting

**DOI:** 10.3389/fphar.2019.01210

**Published:** 2019-10-23

**Authors:** Marta Herrero Fernandez, Raquel Molina Villaverde, Monica Arroyo Yustos, Fatima Navarro Expósito, Jose Luis Lopez Gonzalez, Maria Rosario Luque Infantes, Melchor Alvarez-Mon Soto

**Affiliations:** ^1^Pharmacy Service, Príncipe de Asturias University Hospital, Madrid, Spain; ^2^Diseases of the Immune System and Oncology Service, Príncipe de Asturias University Hospital, Madrid, Spain; ^3^Department of Medicine and Medical Specialties, University of Alcalá de Henares, Madrid, Spain; ^4^Instituto Ramon y Cajal de Investigaciones Sanitarias, IRYCIS, Madrid, Spain

**Keywords:** off-label use, antineoplastic agents, evidence, prevalence, oncology

## Abstract

**Purpose:** The off-label (OL) use of antineoplastic drugs for the treatment of various types of tumors in patients of different disease stages is becoming a common occurrence. The objective of this study was to analyze these patterns by quantification and characterization of the OL use of antineoplastic drugs and their level of scientific evidence in a medium/high-complexity Spanish general university hospital.

**Method:** All oncology patients who underwent OL treatment with one or several antineoplastics during the 10 years from 2002 to 2012 were retrospectively selected. The use of these drugs was considered OL if they were used for indications, stages, lines of treatment, or chemotherapy schemes not reflected in the summary of product characteristics published by the European Medicines Agency at the time of prescription. To calculate the prevalence of patients who received one or more OL treatments during the study period, all patients whose primary or secondary diagnosis had been coded with the diagnoses included in the study were selected through the minimum basic data set (MBDS). This database was cross-referenced with that of the Farmatools^®^ program (Dominion^®^), which collects information on all patients receiving chemotherapy to obtain the total number of patients who received chemotherapy in the hospital during this period.

**Results:** In total, 684 patients and 866 OL treatments were included. The prevalence of patients undergoing OL treatment with antineoplastics was 6%. OL treatments were used mainly for breast, gynecological, lung, and gastric tumors. The most often-used antineoplastic was paclitaxel, followed by gemcitabine, carboplatin, vinorelbine, and capecitabine, which were used mainly in monotherapy and with palliative intent. A total of 56.1% of the OL schemes used had a level of evidence of 2A according to the National Comprehensive Cancer Network, and 55.3% had a level of evidence of 2B according to Micromedex^®^.

**Conclusion:** The OL use of antineoplastics in oncology patients is limited; their use is mainly focused in a small group of tumors and at advanced stages of disease. OL use of antineoplastics occurs under palliative therapeutic strategies with a limited number of drugs, preferably off-patent drugs. In addition, these OL treatments have high levels of clinical evidence.

## Introduction

Cancer is one of the major health problems in countries with developed healthcare systems and is currently the leading cause of death worldwide. Advances and improvements in diagnoses and therapies are contributing to the control and reduction of the death rate from this disease in the United States (USA) and Europe (de Angelis et al., 2014; Ferlay et al., 2015; Organización Mundial de la Salud_OMS, 2018).

Increasing patient survival and quality of life in turn increases the likelihood that patients will receive additional lines of treatment (Hillner and Smith, 2009; Schickedanz, 2010). However, the guidelines and lines of chemotherapy approved by the regulatory agencies are not sufficient to treat the different stages and clinical forms of disease among affected patients. This limitation of the approved therapeutic offerings causes physicians to resort to the use of antineoplastic drugs for conditions that are different from those specified in the product’s technical sheet, which is known as off-label (OL) use.

In clinical practice, OL use in the treatment of various types of tumors and progressing stages of disease is a frequent and relevant reality for patients, prescribing physicians, and the economic cost of the healthcare system. Despite its importance, to date, analyses of the efficacy and efficiency of the OL use of drugs in oncology have been limited. Most of the data available are estimates based on a survey conducted in 1991 by the General Accounting Office (GAO) (Laetz and Silberman, 1991; United States General Accounting Office, 1991) of the USA among oncologist members of the American Society of Clinical Oncology (ASCO). According to the results, more than half of the patients (56%) underwent OL treatment with at least one drug, and 33% of all drugs administered were under conditions other than those specified on the data sheet. In 2007, the ASCO and the European Society for Medical Oncology (ESMO) reported that approximately 50% of the use of antineoplastics was for indications that were not reflected on the data sheet (American Society of Clinical Oncology, 2006; Casali and Executive Committe of ESMO, 2007). According to the National Comprehensive Cancer Network (NCCN®) estimate, in the USA, 50%–75% of all uses of antineoplastics in oncology are OL (Benson and Brown, 2008; Cohen et al., 2009).

Several descriptive studies of OL use in oncology have been performed, such as a study conducted by Levêque et al. (2005) in France, which estimated an annual prevalence of 6.7% by analyzing OL prescriptions of 10 antineoplastic drugs for 10 tumor types. In Switzerland, Joerger et al. (2014) determined that 27.2% of antineoplastic administrations were OL, and a study by Mellor et al. (2009) in Australia reported that 35% of the prescriptions were OL and that the prevalence increased from 22% in 2001 to 35% in 2008.

The present study analyzes the OL use of any antineoplastic agent for patients treated over 10 years. We attempted to minimize the transitory impact of periods related to the upcoming approval of a specific drug by analyzing a long period of time. This approach also allowed us to evaluate OL use in all tumors without limiting the analysis to neoplasms. The study was conducted in an oncology service in the setting of a medium/high-complexity general university hospital. The pattern of OL use was analyzed by quantification and characterization according to the tumor type and stage and the progressive clinical phase of the patient. We also investigated the level of scientific evidence that supports OL use in oncology.

## Materials and Methods

This observational and retrospective study was conducted at the Prince of Asturias University Hospital located in Alcalá de Henares (Madrid, Spain) with 500 beds, specialized diagnosis and treatment units, and more than 10 highly differentiated clinical specialties, which offers healthcare coverage to a population of approximately 400,000 inhabitants. This study analyzed the period between January 1, 2002, and December 31, 2012.

All patients with OL use of one or several antineoplastic drugs, either as a single agent or in combination, during the study period were included. The use of these drugs was considered OL if they were used in indications, stages, lines of treatment, or chemotherapy schemes that were not reflected in the summary of product characteristics published by the European Medicines Agency at the time of prescription. We excluded patients who received treatments with experimental drugs or drugs unauthorized in Spain and those uses that were considered OL due to their use in posologies or routes of administration other than those authorized. Each patient was counted only once; in the cases of patients with more than one OL use of a drug, each treatment was counted separately.

The clinical histories and the dispensing data of the selected patients were reviewed. Information on each patient’s age, sex, Eastern Cooperative Oncology Group (ECOG) status, diagnosis, tumor stage, metastasis, line of treatment, OL drug, type of OL use (monotherapy or combination), patent status and route of administration of the drug, and whether the treatment had curative or palliative intent was collected. To evaluate the status of the patent, we defined a drug “not protected by the patent” if it had expired before or during the study period. We examined the level of evidence of the chemotherapy schemes with OL use (CSOLs) according to the NCCN v1.2015 and Micromedex® 2015 compendia.

The diagnoses were classified according to the ICD.9.10.MC (Ministerio de Sanidad and Servicios Sociales e Igualdad, 2014) classification and were grouped into 14 primary tumor locations or types. A miscellaneous group was created for OL use in rare tumors and those with an absolute frequency lower than 5. A “*patent-protected drug*” was defined as a drug that, at the end of the study (December 31, 2012), was still under a valid patent.

The types of OL use that were classified as monotherapy were additionally classified as “*not indicated for the tumor*” if the drug did not have any indication for that tumor type or in any of the stages of the tumor or as “*indicated in combination*” if the drug had an indication for a certain tumor type in combination with other agents. Likewise, for OL uses classified as combination therapy, those “*with other indications*” were distinguished if they were indicated for that tumor type as monotherapy in another line of treatment or in combination with other agents and as “*without indication*” when at least one drug in the combination had no indication for that type of tumor.

To calculate the prevalence of patients who received one or more OL treatments during the study period, all patients whose *primary or secondary diagnosis* had been coded with the diagnoses included in the study were selected through the minimum basic data set. This database was cross-referenced with that of the Farmatools® program (Dominion®), which collects information on all patients receiving chemotherapy to obtain the total number of patients who received chemotherapy in the hospital during this period.

### Statistical Analysis

A descriptive statistical analysis of the data was conducted. Conformity tests of the categorical variables related to the characteristics of the patients and the OL use of antineoplastic agents (tumor location, drug used, type of OL, intention of treatment, and patent status of the drug) were performed by comparing the proportion of the sample using a chi-square test. A value of p ≤ 0.05 was considered significant. All statistical analyses were performed with SPSS version 18.0 (SPSS Inc., Chicago, IL, USA).

The study was approved by the Ethics Committee for Scientific Research at Prince of Asturias University Hospital and was classified by the Spanish Agency for Medicines and Health Products as post-authorization study with designs other than prospective follow-up (EPA-OD).

## Results

### Demographic and Clinical Characteristics of the Sample With Oncological Disease

**Figure 1:**
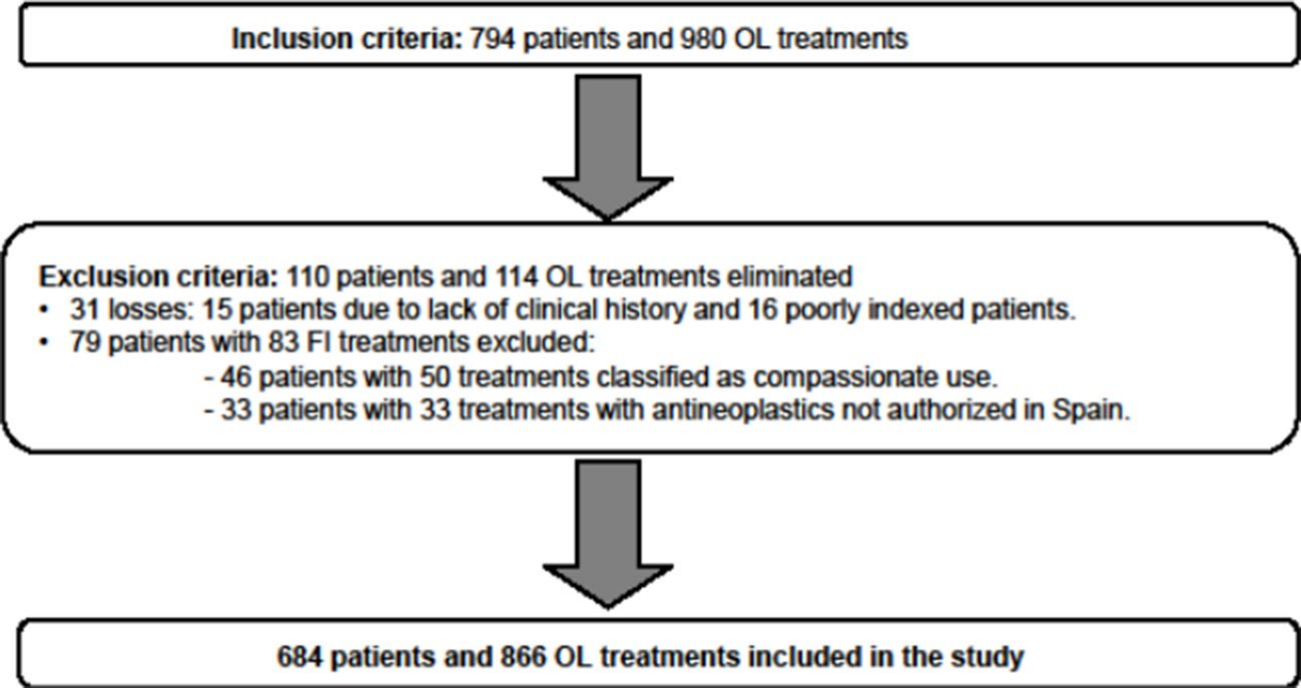
Inclusion or exclusion of patients from the study according to the selection criteria.

This study included 794 patients with oncological disease and 980 OL treatments during the 10 years of the study. After the exclusion criteria were applied to this sample, 684 patients and 866 OL treatments were included in the analysis (**Figure 1**).

The study showed a significantly higher amount of OL use in women with an average age of less than 65 years and good general condition (p < 0.001). OL use was predominant in patients with stage IV disease and the presence of metastasis (p < 0.001) (**Table 1**).

**Table 1:** General characteristics of patients who received off-label treatments.

Patient characteristics	N = 866	%
Gender*		
Male	370	42.7
Female	496	57.3
Age*		
Mean age of patients (years)	58.21 ± 11.57	
Age <65	601	69.4
Age ≥65	265	30,6
Performance status (ECOG)*		
0	402	46.4
1	308	35.6
2	156	18
Presence of metastatic disease*		
Yes	684	79
No	182	21
Disease stage*		
I	39	4.5
II	59	6.8
III	84	9.7
IV	684	79
**Previous antineoplastic treatment line**		
0-3	704	81.3
3-6	142	16.4
6-9	19	2.2
>9	1	0.1
*p < 0.001.		

### OL treatment Is Preferably Used in Oncology for a Small Group of Tumor Types and With Limited Use of Agents

Among all cancer patients treated with chemotherapy (n = 11,385), the prevalence of those receiving OL use of antineoplastic drugs was 6% (684/11,385).

OL treatments were used mainly in four locations or tumor types: breast cancer (25.2%), gynecological tumors (16.1%), lung cancer (11.8%), and gastric cancer (10.2%). The OL use in these tumor types was significantly greater than that in all other tumor types (63.3% vs 36.7%, respectively; p < 0.001) (**Table 2**).

**Table 2:** Primary location of tumors involved in off-label use of antineoplastic drugs.

Tumor location	Treatment (n = 866)	%
Breast	218	25.2
Gynecological	139	16.1
Lung	102	11.8
Gastric	88	10.2
Head and neck	69	8
Biliopancreatic	64	7.4
Colorectal	53	6.1
Soft tissue sarcoma	29	3.3
Bladder-urothelial	25	2.9
Prostate and testicle	24	2.8
Melanoma	22	2.5
Esophagus	15	1.7
Miscellaneous	12	1.4
Liver	6	0.7

We found an OL use of 27 antineoplastic agents, and the most commonly used was paclitaxel (19.2%), followed by gemcitabine (10.9%), carboplatin (9.6%), vinorelbine (8.8%), and capecitabine (8.7%). The OL use of these five drugs was significantly more frequent than that of the other 22 drugs (57.2% vs 42.8%, respectively; p < 0.001) and was essentially focused on the four most frequent tumor types. The OL use of drugs as palliative treatment predominated over their use as curative treatment (79% vs 21%, p < 0.001) (**Table 3**).

**Table 3:** Characteristics of treatments with off-label use of antineoplastics.

Drug	Treatment (n = 866)	%
Paclitaxel	195	19.2
Gemcitabine	111	10.9
Carboplatin	98	9.6
Vinorelbine	89	8.8
Capecitabine	88	8.7
Irinotecan	77	7.6
Trastuzumab	74	7.3
Oxaliplatin	71	7
Docetaxel	54	5.3
Bevacizumab	36	3.5
Temozolomide	22	2.2
Liposomal doxorubicin	19	1.9
Cetuximab	16	1.6
Etoposide	13	1.3
Lapatinib	11	1.1
Cisplatin	8	0.8
Topotecan	8	0.8
IL-2	5	0.5
Pemetrexed	5	0.5
Adriamycin	4	0.4
Albumin-paclitaxel	4	0.4
Pegylated liposomal doxorubicin	3	0.3
Nilotinib	2	0.2
Cyclophosphamide	1	0.1
Imatinib	1	0.1
Sorafenib	1	0.1
Sunitinib	1	0.1
**Type of off-label use**		
Monotherapy	609	70.3
Not indicated for the tumor	458	52.9
Indicated in combination	151	17.4
Combinations	257	29.7
With other indications	126	14.6
Without indication	131	15.1
**Intent of off-label treatment**		
Curative	182	21
Palliative	684	79
**Patent status**		
Patent protected	235	27.1
Off-patent	631	72.9
**Line of off-label treatment**		
1ª line	150	17.3
2ª line	243	28.1
3ª line	197	22.7
4ª line	114	13.2
Successive lines	162	18.7

The wide OL use of paclitaxel was due to its use in monotherapy in nonsmall cell lung cancer; cancers of the cervix, bladder, and esophagus; and head and neck tumors. The OL use of gemcitabine was notable as a single agent in breast and ovarian tumors, for which it has an indication when combined with other agents. OL use of carboplatin occurred for breast cancer, both as monotherapy and combined with other agents. The OL use of vinorelbine was due to its use as a single agent for the treatment of gynecological and prostate tumors; the OL use of capecitabine mainly included its use in a neoadjuvant form in rectal cancer and as an adjuvant in high-risk stage II colon cancer and with palliative intent in pancreatic cancer.

Most of the drugs used were not protected by patents (73% vs 27%; p < 0.001). The most commonly used route of administration was intravenous. Regarding the line of treatment for OL use of antineoplastics, no significant differences were observed between the first four lines used. However, 50.8% of OL uses were as second- and third-line treatments.

The OL treatments were mainly used in monotherapy (70.3% vs 29.7%; p < 0.001), with a notable use of drugs without any type of indication approved for the tumor on which they were used (52.9%). **Table 4** indicates the type of OL use for each tumor site.

**Table 4:** Type of off-label use for each location/tumor type.

Location/Tumor type	Type of Label Treatment							
	Monotherapy				Combination			
	Not indicated for the tumor		Indicated in combination		Without indication		With other indications	
	Patients (n)	(%)	Patients (n)	(%)	Patients (n)	(%)	Patients (n)	(%)
Biliopancreatic								
*Pancreas*	46	86.8	0	0	7	13.2	0	0
*Biliary*	10	90.9	0	0	1	9.1	0	0
Bladder-urothelial	24	96	0	0	1	4	0	0
Breast	27	12.39	41	18.81	46	21.1	104	47.7
Colorectal								
*Colon*	17	77.3	0	0	5	22.7	0	0
*Rectal*	30	96.8	0	0	1	3.2	0	0
Esophagus	15	100	0	0	0	0	0	0
Gastric	70	79.6	17	19.3	1	1.1	0	0
Gynecological								
*Cervix*	22	66.7	0	0	11	33.3	0	0
*Endometrium*	5	23.8	0	0	16	76.2	0	0
*Ovary*	43	50.57	32	37.63	10	11.8	0	0
Head and Neck	37	73.9	14	20.3	18	26.1	0	0
Liver	6	100	0	0	0	0	0	0
Lung								
*Microcytic*	28	87.5	4	12.5	0	0	0	0
*Not microcytic*	5	7.15	43	61.45	0	0	22	31.4
Melanoma	17	77.3	0	0	5	22.7	0	0
Miscellaneous								
GIST (gastrointestinal stromal tumor)	2	100	0	0	0	0	0	0
*Neuroendocrine*	0	0	0	0	2	100	0	0
*Osteosarcoma*	0	0	0	0	1	100	0	0
*Kidney*	1	100	0	0	0	0	0	0
*Adrenal*	1	100	0	0	0	0	0	0
*Thymoma*	2	66.7	0	0	1	33.3	0	0
TOD (tumor of unknown origin)	2	100	0	0	0	0	0	0
Prostate and Testicle								
*Prostate*	11	100	0	0	0	0	0	0
*Testicle*	12	100	0	0	0	0	0	0
Soft tissue sarcoma	24	82.8	0	0	5	17.2	0	0

### OL Treatments Are Mostly Supported by Clinical Evidence

The analysis of the degree of evidence of the 114 CSOLs included in the study showed the following: the NCCN^®^ indicated a 2A level of evidence for 56.1% of the schemes, while the Micromedex® indicated that practically the same percentage (55.3%) of schemes had a 2B level of evidence. The same analysis was performed on the 866 OL treatments included in the study; in this case, 64% of the treatments used had a 2A level of evidence according to the NCCN® and a 2B level of evidence according to Micromedex®.

The percentages of CSOLs that were not reflected in either of the two compendia were 22.8% in NCCN® and 34.2% in Micromedex® however, these CSOLs accounted for only 11.6% and 19.4% of 866 OL treatments analyzed, respectively (**Table 5**).

**Table 5:** Evidence for off-label treatment.

	Off-label chemotherapy regimens n = 114	%	Treatments n = 866	%
**NCCN^®^**				
1	6	5.3	113	13
2A	64	56.1	554	64
2B	16	14	104	12
3	2	1.8	3	0.3
N/A	26	22.8	92	10.6
***MICROMEDEX*** ^®^				
1	1	0.9	30	3.5
2A	8	7	101	11.7
2B	63	55.3	555	64.1
3	3	2.6	6	0.7
N/A	39	34.2	174	20.1

N/A (Not applicable): chemotherapy regimens for which evidence is not included in the compendium that was evaluated.

## Discussion

The results of this study showed that in the setting of a general university hospital, the OL use of antineoplastic drugs in patients with tumor pathology is limited and is focused on a small group of tumors (breast, gynecological, lung, and gastric) in patients under 65 years old with good general condition and advanced-disease stages. The OL use of antineoplastic drugs mainly occurs in a palliative therapeutic strategy with a limited number of drugs, preferably off-patent drugs. In addition, these OL treatments attain high levels of clinical evidence.

The OL use of antineoplastic drugs in oncology is a therapeutic practice that is used in healthcare in a highly variable manner according to the published studies. Such studies have specifically analyzed the prescriptions for patients with specific tumors without specifying whether they are different patients or successive lines used in the same patient. This fact can explain the variability observed in the reflected prevalence of OL use—between 6% and 35% (Leveque et al., 2005; Mellor et al., 2009; Joerger et al., 2014). Our results of the analysis of all patients who received OL antineoplastic treatment show that the frequency of use of this therapeutic strategy is limited and is focused on 6% of patients. These data contrast with those obtained in the GAO survey conducted on a sample of North American medical oncologists (Laetz and Silberman, 1991; United States General Accounting Office, 1991). These differences can be explained, in addition to the methodological reasons previously described, by other reasons, including accessibility and control of the OL use of antineoplastic drugs in the setting in which this study was conducted: a hospital of a national universal healthcare system for the population served by the center with free access by the ill. However, until 2009, the management of requests and approval for the OL use of antineoplastic drugs in our national healthcare system was centralized in a review committee external to the hospital; thus, this management strategy for OL drug use could have influenced the low comparative prevalence found in our study.

To assess the OL treatments used in routine clinical practice, the epidemiology of the tumor and advances in the clinical and therapeutic management of the disease must be taken into account (Hanahan and Weinberg, 2000; Kocs and Fendrick, 2003; Newell, 2005; Hillner and Smith, 2009; Schickedanz, 2010). Likewise, improvements in supportive care have also increased the number of candidates for additional chemotherapy lines (Kocs and Fendrick, 2003). Our study shows that the OL use of antineoplastic drugs is significantly greater in palliative oncological treatment strategies, in metastatic stages of the disease, and especially as second- and third-line treatments. These findings are consistent with those obtained in the USA by the GAO (Laetz and Silberman, 1991; United States General Accounting Office, 1991; Eastman, 2005) on the OL use of antineoplastic agents and those of other studies with smaller sample sizes (Kocs and Fendrick, 2003; Peppercorn et al., 2008; Roila et al., 2009; Cioffi et al., 2012; Cohen et al., 2013; Joerger et al., 2014).

This work indicates that the OL use of antineoplastic drugs is mainly focused on breast cancer, gynecological tumors, lung cancer, and gastric cancer. These four neoplasms share the characteristics of high prevalence and/or mortality (Ferlay et al., 2013; de Angelis et al., 2014; Ferlay et al., 2015; Organización Mundial de la Salud_OMS, 2018; SEOM, 2019). In previous studies, such as that of Joerger et al. (2014), which included 10 tumor types, the OL use of antineoplastics was more prevalent for gastrointestinal, breast, lung, and gynecological tumors. In the study conducted by Leveque et al. (2005), of 10 tumor types, prostate, breast, bladder, and ovarian cancers had the most OL prescriptions.

Our findings indicate that the results obtained by other authors who performed selective analysis of some tumor types included those more prevalent in a general analysis of all tumors (Leveque et al., 2005; Mellor et al., 2009; Joerger et al., 2014).

In our study, OL use was mainly focused on the use of 5 of 27 drugs, including paclitaxel, gemcitabine, carboplatin, vinorelbine, and capecitabine. All five drugs are cytotoxic agents with recognized activity in different tumors, and their use is supported by more and less extensive studies and for advanced pathologies. We found that paclitaxel had the most OL use; in contrast, OL use of docetaxel was limited. These data could be explained by the introduction of generic drugs and the changes observed in toxicity patterns, which could have changed the attitudes toward medical prescription. This possibility could affect the discrepancy between the OL use of docetaxel observed in our study and that found by other authors (Leveque et al., 2005; Joerger et al., 2014). Joerger et al. (2014) examined the OL use of 10 antineoplastics and 437 prescriptions in their analysis and found that OL use was more frequent with paclitaxel, followed by carboplatin and docetaxel. In the study by Levêque et al. (2005), the drug most commonly prescribed for OL use was docetaxel, followed by oxaliplatin, fludarabine, carboplatin, gemcitabine, paclitaxel, and irinotecan. The authors explained that the wide use of docetaxel was mainly due to its use in prostate cancer; in 2002, docetaxel still did not have an indication for this type of tumor in France.

The evaluation of our results on the OL use of antineoplastics in relation to other studies requires consideration of some relevant aspects of oncological treatment. Among them, the analyzed period is notable due to the advances made in the management of neoplasms and the scope of the study, which includes the healthcare system in which the study is conducted, the country, and the hospital level. From a general perspective, the distribution of the OL use of antineoplastics according to the type or tumor location reflected in our study shows patterns of indication and use similar to those described in two previous studies. It should be noted that these three studies were conducted in European healthcare settings.

Notably, the OL use of antineoplastic drugs is more common in monotherapy than in combination therapies, with a predominant use in groups of drugs without any authorized indication for the tumor for which they were used. This finding can be related to the palliative intent of treatment and the use of these drugs as second- and third-line treatments. In this type of clinical situation, an increase in patient survival is sought *via* good control of the disease, with quality of life prevailing over other factors. In addition, the previous lines of treatment will limit the available options according to the health results obtained with the use of these drugs and any toxicities developed during the treatment.

In our analysis, a majority of the drugs used for OL treatments were not protected by patents. This finding is consistent with that of other publications that establish that an increase in OL use may occur among antineoplastic drugs after the patent has expired. This loss of patent could lead to a lack of interest on the part of the pharmaceutical industry to obtain new indications (Radley et al., 2006; Casali and Executive Committe of ESMO, 2007; Roila et al., 2009; Gota and Patial, 2011; Lerose et al., 2012; Wittich et al., 2012; Conti et al., 2013). Likewise, other drugs are not authorized for all indications for which they could be effective, mainly due to the high economic cost and time required to obtain a new indication (Boos, 2003; Gupta and Nayak, 2014).

Currently, several authors have established that in clinical practice, a chemotherapy scheme is supported by evidence and therefore is “*medically accepted*” if it is considered category 1 or 2A by the NCCN compendium or class 1, 2A, or 2B by Micromedex® (American Society of Clinical Oncology, 2006; Benson and Brown, 2008; Mehr, 2012). Independent clinical investigations, despite being considered of lower scientific quality than clinical trials, generate experience and provide data on the efficacy and safety of OL use of antineoplastics (Gazarian et al., 2006; Casali and Executive Committe ESMO, 2007; Mellor et al., 2009; Feliu and Espinosa, 2013; Gupta and Nayak, 2014).

Despite the debate that exists regarding the evidence supporting the OL use of antineoplastic drugs (Gazarian et al., 2006; Abernethy et al., 2009; Irwin et al., 2012; Pfister, 2012), our results indicate that most of these OL uses are performed with significant scientific support, such as that collected in the North American therapeutic compendia. This coincides with studies previously published by Gota and Patial (2011) and Mellor et al. (2012) that used the NCCN to assess the degree of evidence of the OL schemes analyzed. Regarding the OL use of antineoplastic drugs that were not included in these compendia, drugs or regimens used in marginal cases were included, in which the justification for their use is based on results published in scientific journals without having reached the levels of so-called recognized therapeutic clinical evidence. It should also be noted that the compendia used originated from the USA, while the usual clinical practice was performed in Europe, and the approved indications for each drug are different between the two continents.

Our analysis presents some important limitations such as a one-center study, and therefore, the results focus on the type of population that our hospital serves, and epidemiologically infrequent or “rare” tumors do not reach a relevant qualitative significance. Multicentric studies should be carried out to confirm our results and to analyze the use of OL antineoplastics in less frequent tumors.

## Data Availability Statement

All datasets generated for this study are included in the article/Supplementary Files.

## Ethics Statement

The studies involving human participants were reviewed and approved by Comite Etico de Investigacion Clinica del Hospital Universitario Principe de Asturias. The ethics committee waived the requirement of written informed consent for participation.

## Author Contributions

MF designed the study, performed the experiments, analyzed the data, and wrote the manuscript. RV designed the study, performed the experiments, and wrote the manuscript. MY performed the experiments. FE performed the experiments. JG performed the experiments. MI performed the experiments. MA designed the study and wrote the manuscript.

## Funding

This study was partially supported by a grant from Comunidad Autónoma de Madrid (MITIC B2017/BMD-3804).

## Conflict of Interest

The authors declare that the research was conducted in the absence of any commercial or financial relationships that could be construed as a potential conflict of interest.

## Abbreviations

OL, off-label; SmPC, summary of product characteristics; CSOLs, chemotherapy schemes with off-label use; MBDS, minimum basic data set; AEMPS, Spanish Agency for Medicines and Health Products.
